# A Chlorophyll-Derived Phylloxanthobilin Is a Potent Antioxidant That Modulates Immunometabolism in Human PBMC

**DOI:** 10.3390/antiox11102056

**Published:** 2022-10-19

**Authors:** Cornelia A. Karg, Lucia Parráková, Dietmar Fuchs, Harald Schennach, Bernhard Kräutler, Simone Moser, Johanna M. Gostner

**Affiliations:** 1 Department of Pharmaceutical Biology, Ludwig-Maximilian University of Munich, Butenandtstr. 5–13, 81977 Munich, Germany; 2 Institute of Medical Biochemistry, Medical University of Innsbruck, Innrain 80, 6020 Innsbruck, Austria; 3 Institute of Biological Chemistry, Medical University of Innsbruck, Innrain 80, 6020 Innsbruck, Austria; 4 Central Institute of Blood Transfusion and Immunology, University Hospital, Anichstr. 35, 6020 Innsbruck, Austria; 5 Institute of Organic Chemistry, Center for Molecular Biosciences, University of Innsbruck, Innrain 80/82, 6020 Innsbruck, Austria

**Keywords:** natural products, tetrapyrroles, phyllobilin, tryptophan, kynurenine

## Abstract

Phyllobilins are natural products derived from the degradation of chlorophyll, which proceeds via a common and strictly controlled pathway in higher plants. The resulting tetrapyrrolic catabolites—the phyllobilins—are ubiquitous in nature; despite their high abundance, there is still a lack of knowledge about their physiological properties. Phyllobilins are part of human nutrition and were shown to be potent antioxidants accounting with interesting physiological properties. Three different naturally occurring types of phyllobilins—a phylloleucobilin, a dioxobilin-type phylloleucobilin and a phylloxanthobilin (PxB)—were compared regarding potential antioxidative properties in a cell-free and in a cell-based antioxidant activity test system, demonstrating the strongest effect for the PxB. Moreover, the PxB was investigated for its capacity to interfere with immunoregulatory metabolic pathways of tryptophan breakdown in human blood peripheral mononuclear cells. A dose-dependent inhibition of tryptophan catabolism to kynurenine was observed, suggesting a suppressive effect on pathways of cellular immune activation. Although the exact mechanisms of immunomodulatory effects are yet unknown, these prominent bioactivities point towards health-relevant effects, which warrant further mechanistic investigations and the assessment of the in vivo extrapolatability of results. Thus, phyllobilins are a still surprisingly unexplored family of natural products that merit further investigation.

## 1. Introduction

Plants must maintain and protect themselves in a changing environment as they lack the flight response. As a result, sophisticated strategies have evolved, producing a broad array of natural products that support stress resistance. It is no surprise that such bioactive molecules are the focus of biomedical research [1]. The de-greened products from the breakdown of the green plant pigment chlorophyll (Chl) have, in fact, joined the group of highly interesting bioactive compounds. Considering the ever-puzzling and obvious disappearance of Chl from the biosphere, estimated to amount to about one billion tons every year on earth [2], the stable degradation products of Chl represent an enormous reservoir of natural products. Chl breakdown is not only a sign of senescence in autumn, it also plays a role in plants’ response to stress [3]. Hence, the discovery of its degradation product adds an important puzzle piece to understand one of the most fundamental processes of life. The first such Chl-degradation product, now classified as a phyllobilin (PB) due to the structural similarity to bilins [4], was discovered rather recently, its characterization being accomplished only roughly 30 years ago [5]. The structure of this colorless catabolite from senescent leaves of barley gave the first hints to the changes that happen to Chl during its degradation: it is a linear tetrapyrrole devoid of the Mg ion, and carries a range of hydrophilic modifications [6]. 

**Scheme 1:**
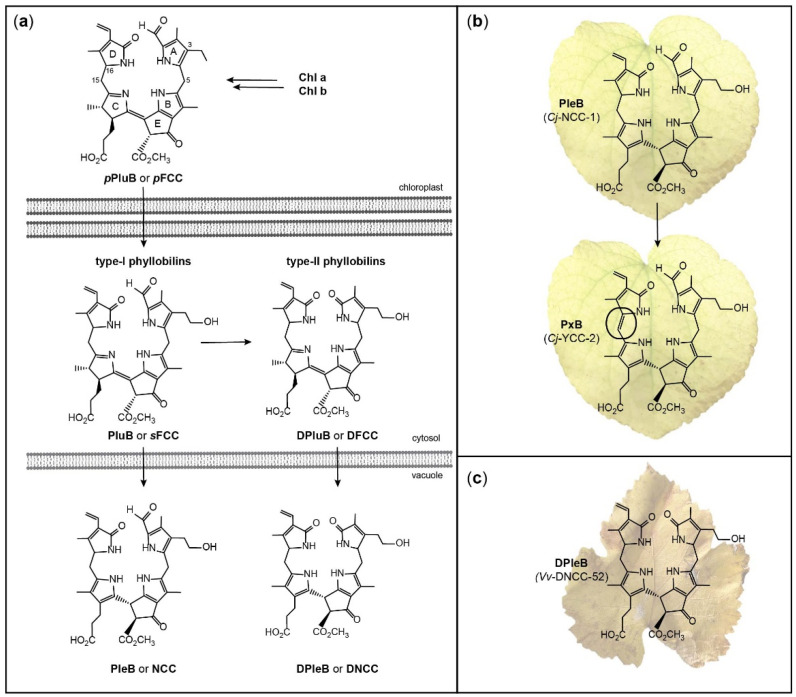
(**a**) Catalyzed reaction to the DPluB, introducing the second branch of type-II PBs. Modified PluBs (PluBs and DPluBs) are transported into the vacuoles and converted into nonfluorescent Chl catabolites, PleBs or DPleBs. (**b**) Structures of the major PleB (*Cj*-NCC-1) from senescent leaves of *Cercidiphyllum japonicum* and the corresponding oxidation product, the PxB (*Cj*-YCC-2), generated in plants by a still elusive ‘oxidative activity’. (**c**) Structure of the type-II PB, the DPleB (*Vv*-DNCC-52) from senescent leaves of grapevine (*Vitis vinifera*).

The degradation of Chl has been shown to be a strictly controlled multistep pathway with fundamental steps being common to all higher plants [7]. Its primary purpose is considered to be a detoxification by eliminating the phototoxic properties of Chl [8]. This so called PaO/phyllobilin pathway, the biochemistry of which has been the focus of intense studies [9], achieves the removal of the hydrophobic phytyl side chain and of the Mg-ion, followed by the cleavage of the macrocycle by pheophorbide a oxygenase (PaO) and reduction of the so formed ring-opened tetrapyrrole by red Chl-catabolite reductase (RCCR) to the primary fluorescent Chl catabolite (*p*FCC), the common key intermediate [10,11,12], also named here the primary phyllolumibilin (*p*PluB). At this stage, the pathway of Chl degradation splits into two major branches, as known so far: modified PluBs that carry the characteristic formyl group at ring A of the molecule (hence type-I PBs), are transported into the vacuoles. Due to the acidic pH in the vacuoles, PluBs are non-enzymatically isomerized to nonfluorescent Chl catabolites (NCCs) or phylloleucobilins (PleBs) (Scheme 1a) [13,14,15]. PleBs are non-phototoxic, polar type-I PBs, and were the first linear tetrapyrrolic catabolites derived from Chl to be identified. In the other major branch of the pathway that generates the type-II PBs, a cytochrome P450 removes the aldehyde moiety forming dioxobilin-type PluBs (DPluBs) with a lactam group at ring A, which then undergo a similar isomerization in the vacuoles to the corresponding DPleBs (earlier classified as DNCCs) (Scheme 1a) [16,17]. Remarkably, DPleBs are the most abundant catabolites in *Arabidopsis thaliana*, where they account for over 90% of all identified ‘late’ stage catabolites [18,19]. Furthermore, DPleBs (DNCCs) have been identified in leaves of Norway maple and grapevine (Scheme 1c) [20,21].

In the later phase of the degradation pathway, oxidation products of PleBs and DPleBs can be observed in extracts of senescent leaves. These oxidation products were found to be colored; yellow Chl catabolites (YCCs), also called phylloxanthobilins (PxBs), are directly formed from PleBs by introducing a double bond in the ring system, accounting for the yellow color (Scheme 1b) [13]. The discovery of the first PxB in senescent leaves of *Cercidiphyllum japonicum* ((Katsura tree) and then called a YCC) [22] paved the way for identifying more PxB structures, among others in leaves of lime tree [23], waterweed (*Egeria densa*) [24], and wych elm [25]. Interestingly, the identification of PxBs in an unprecedented amount and diversity in leaf extracts of the medicinal plant *Echinacea purpurea* (purple coneflower) [26] as well as the identification of PxBs in leaves of spinach and *Spathiphyllum wallisii* (peace lily), for which only PleB structures were reported before [27,28], shows especially that PxBs appear to be more abundant catabolites than previously assumed [29]. Moreover, PxBs are structurally similar to the heme degradation product bilirubin [13], a natural product with antioxidative properties, which caught attention in terms of investigating possible physiological effects. In contrast to the heme degradation pathway, Chl degradation yields a striking diversity of catabolites, with more and more structural modifications being discovered [30]. Although the observed high diversity of catabolites appears to be less relevant for the breakdown process [31], it calls for investigations of bioactivities related to human health.

So far, a great variety of phytochemicals have been analyzed for their potential to interfere with key signaling pathways in the human system. Major attention was payed to phytochemical antioxidants as the attenuation of the oxidative stress is not only related to cytoprotection but often also to anti-inflammatory activities, as redox biochemistry is crucially involved in the orchestration of immunological cascades [32]. Phytochemical antioxidants are either classical reducing agents, radical scavengers and/or indirectly enhance intracellular antioxidative capacities by interfering with cellular antioxidative systems or by upregulation of protective pathways [33]. Whether a compound exerts antioxidant effects in vivo truly also depends on its bioavailability and the chemical microenvironment at the side of action. In addition, metabolic steps through commensal bacteria have to be taken into account besides other ADME parameters [32]. Still, in vitro methods are helpful in the assessment of these properties. 

In particular, the inflammation-induced breakdown of the essential amino acid L-tryptophan (Trp) along the kynurenine (Kyn) axis has emerged as a useful target to study immunomodulatory properties of (phyto)chemicals in vitro, using human peripheral blood mononuclear cells (PBMC) as a cell model in which the monocyte/T-cell crosstalk is maintained [34,35]. Indoleamine 2,3-dioxygenase 1 (IDO-1), known to be a key immunoregulatory enzyme, is upregulated under inflammatory conditions and catalyzes the breakdown of Trp to *N*-formylkynurenine in the first and rate limiting step of the kynurenine pathway. In addition to protein synthesis, the dietary tryptophan, which is available for the formation of other molecules, is largely metabolized along the kynurenine axis already under physiological conditions and this route is even more used in a (pro)inflammatory situation [36]. The Trp–Kyn pathway is an essential component of the T helper 1 (Th-1)- type cellular immune response and of high relevance as biomarker in several human disorders associated with an activated immune system e.g., infections and malignancies [37].

In the current study, we focused on three central aspects to investigate the bioactivities of PBs: Their potential to counteract different types of oxidative stress both in cell models and in cell-free environments, and the capacity to interfere with Trp breakdown in PBMC. The latter is an immunoregulatory pathway, which is widely in use to screen for immunomodulatory capacities of antioxidants [34]. With the aim of comparing bioactive properties of PBs that basically differ in only minor structural features, we selected three candidate molecules for our experiments: a PleB from the Katsura tree (*Cercidiphyllum japonicum, Cj*-NCC-1) and the corresponding oxidation product, a PxB (*Cj*-YCC-2), which differ in one double bond at C15/C16 (Scheme 1b). Furthermore, we included a DPleB isolated from leaves of the grape vine (*Vitis vinifera, Vv*-DNCC-52) in our experiments (Scheme 1c), which carries a γ-lactam group at ring A in contrast to the PleB from Katsura, which features a formyl group. All structures share the identical ‘simple’ modification motif of PBs, carrying a hydroxyethyl group at ring A and a vinyl group at ring D [38]. 

## 2. Materials and Methods

### 2.1. General

HPLC grade acetonitrile (MeCN) and methanol (MeOH) were from VWR (West Chester, USA), ultra-pure water (18 MΩ.cm^−1^) from a Millipore apparatus, potassium phosphate monobasic (KH_2_PO_4_), puriss. p.a., potassium phosphate dibasic (K_2_HPO_4_), puriss. p.a., and ammonium acetate, puriss. p.a. were from Fluka (Buchs, CH). 1 g SepPak C18 cartridges were from Waters Associates (Milford, CT, USA). Media and chemicals were obtained from Sigma-Aldrich (Vienna, Austria) if not stated otherwise. 

pH-measurements: WTW Sentix 21 electrode connected to a WTW pH 535 digital pH meter.

### 2.2. Plant Material

*Cercidiphyllum japonicum* leaves were harvested from trees located in the Botanical Garden of the University of Innsbruck (Innsbruck, Austria, 47°16′03.2″ N, 11°22′43.9″ E) and stored at −80 °C in a deep freezer. Isolated PleB and PxB were compared with compounds isolated from *Cercidiphyllum japonicum* leaves that were collected in the Maria-Ward-Straße, Munich (48°09′41.0″ N 11°29′58.8″ E), identified by S. Renner (Department of Systematic Botany and Mycology, Faculty of Biology, University of Munich), and of which a voucher specimen (Moser & Karg 3) has been deposited in the Munich herbarium (acronym M). 

Senescent grapevine leaves were collected on 14 November 2014 from healthy chardonnay grapevine plants in an experimental vineyard (“Piglon”), at Laimburg Research Centre (Pfatten/Vadena, South Tyrol, Italy). The grapevines, planted in 2006, were grown on a Guyot training system and managed according to the integrated production guidelines. The collected leaves were immediately frozen at −80 °C, transported frozen to Innsbruck and stored at −80 °C until further use.

### 2.3. Chromatography

Analytical HPLC: Dionex Summit HPLC system with manual sampler and online degasser, P680 HPLC pump, UVD340U diode array detector and a Rheodyne injection valve with a 200 µL, 1.8 mL or 2.0 mL loop; column Phenomenex Hyperclone ODS 5 µm 250 × 4.6 mm i.d., with Phenomenex ODS 4 × 3 mm i.d. pre-column; mobile phase A = ammonium acetate buffer 10 mM pH 7, B = MeCN; solvent composition: 0–2.5 min 17% B, flow 0.8 mL/min; 2.5 to 2.7 min 17% B, flow reduced to 0.5 mL/min; 2.7–25 min 17% to 45% B, flow 0.5 mL/min. Data were processed with Chromeleon 6.50.

### 2.4. Spectroscopy

UV/Vis: λmax [nm] (ε_rel_), Hitachi U-3000 spectrophotometer, concentrations of PBs were calculated using Lambert–Beer’s law and the extinction coefficients of log ε = 4.23 at λmax = 312 nm (for PleB [39]), log ε = 4.51 at λmax = 426 nm (for PxB [22]), and log ε = 4.49 at λ = 237 nm (shoulder, for DPleB [21]).

ESI-MS: *m*/*z* (relative abundance, type of ion), signals due to isotopomers and their relative intensities are shown for [M+H]^+^; Thermo LTQ XL or LTQ Orbitrap (for high resolution MS) mass spectrometer, ESI source, positive ion mode, spray voltage 4.0 kV, data were processed with Xcalibur.

### 2.5. Isolation and Preparation of Phyllobilins

Isolation of PleB and PxB from senescent leaves of *Cercidiphyllum japonicum* was performed as published previously [29]. Pure PxB was crystallized according to the protocol of Li et al. [40]. DPleB was isolated from yellow grapevine leaves following the published protocol [20]. 

All isolated compounds were either stored at −20 °C and dissolved in 0.2% (*v*/*v*) ethanol in HBSS prior use or stored as DMSO stock at −20 °C until further use. 

### 2.6. Cell Culture and Isolation of PBMC

All cells were cultured at 37 °C in a humidified atmosphere containing 5% CO_2_. The human colon adenocarcinoma-derived cell line Caco-2 (American Type Culture Collection (ATCC), Manassas, VA, USA) was grown in MEM with Earle’s salts supplemented with 1% (*v*/*v*) 200 mM L-glutamine, 10% (*v*/*v*) heat-inactivated fetal bovine serum (FBS) (all from GIBCO, Thermo Fisher Scientific, Dreieich, Germany), 1% (*v*/*v*), non-essential amino acids and 2.5% (*v*/*v*) 1 M HEPES (*N*-2-hydroxyethylpiperazine-*N*-2-ethane sulfonic acid). Human peripheral mononuclear cells (PBMC) were isolated from whole blood obtained from healthy donors of whom informed consent was obtained that their donated blood might be used for scientific purposes, at the Central Institute of Blood Transfusion and Immunology, University Hospital, Innsbruck, Austria. The local ethics committee confirmed that no further approval is required for using anonymized leftover specimens from blood donations of the local blood bank for scientific purposes; the study was performed according to the Helsinki declaration. Separation of blood cells was performed by density centrifugation using Lymphoprep separation medium as reported elsewhere [35]. PBMC were maintained in RPMI 1640 medium supplemented with 10% (*v*/*v*) heat-inactivated FBS, 1% (*v*/*v*) 200 mM L-glutamine (both from GIBCO, Thermo Fisher Scientific, Germany) and gentamicin was added at a final concentration of 50 µg/mL.

### 2.7. Stability of PxB in Medium 

Cells were plated on Petri dishes in media containing PxB (45 µM), and incubated at 37 °C. After 48 h, the supernatant was removed, cells were harvested with methanol and lysed by repeated liquid nitrogen freeze/vortex cycles. The cell lysate was centrifuged at 13 krpm for 3 min, and the clear solution was used. Both samples (lysate and cell supernatant) were desalted over a SepPak cartridge. The samples were analyzed by analytical HPLC. Fractions were collected from the analytical HPLC on ice and directly analyzed by ESI-MS.

### 2.8. Oxygen Radical Absorbance Capacity (ORAC) Assay

The oxygen radical absorbance capacity (ORAC) assay was applied to assess in vitro antioxidant activities by determining the PBs‘ capacity to scavenge peroxyl-radicals [41]. The assay was performed as described previously by Gostner et al. [42] with minor modifications. In brief, fluorescein (6.3 × 10^−8^ M; AnaSpec, San Jose, CA, USA) dissolved in 75 mmol/L phosphate buffer (pH 7.4) was used as a probe, then 2,2′-azobis (2-amidinopropane) dihydrochloride (AAPH; Wako Chemicals, Osaka, Japan) was added as a peroxyl radical generator (final concentration of 1.25 × 10^−2^ M). The vitamin E derivate Trolox was used as a reference compound (dissolved in 75 mmol/L phosphate buffer) in a concentration range of 0.78 to 6.25 µmol/L. Dilutions in potassium phosphate buffer pH 7 of PleB (19.1 µM), PxB (12.3 µM), and DPleB (15.6 µM) were prepared from dimethyl sulfoxide (DMSO) stocks. Epigallocatechin-gallate (EGCG) and vitamin C (both diluted in potassium buffer from DMSO stocks) were used as controls. 

The plate was incubated for 30 min at 37 °C prior to the addition of AAPH. The fluorescence of fluorescein (480 nm/530 nm) was recorded by a Tecan infinite F200 PRO plate reader (Tecan Group Ltd., Männedorf, Switzerland) every 70 s after the addition of AAPH for 36 cycles at 37 °C. All measurements were expressed relative to the initial reading. Final results were calculated using the differences of areas under the fluorescein decay curves (AUC) between the blank and a sample [41]. The results were expressed as Trolox equivalents (TE; µmol TE/µmol sample).

### 2.9. Intracellular ROS Measurement 

The influence of test substances on intracellular reactive oxygen species (ROS) levels was measured by using 2′,7′-dichlorofluorescein diacetate with a modified protocol of Bass et al. [38,43]. Caco-2 cells were seeded in 96-well plates (2 × 10^5^ cells/100 µL/well). After 24 h, cells were washed with phosphate-buffered saline (PBS), incubated with 50 µL/well of 25 µM DCFH-DA dissolved in Hank’s buffered salt solution (HBSS) and light protected at 37 °C for 1 h. Then cells were treated with either increasing concentrations of PBs, quercetin as a control (10 µM), HBSS as a buffer control or the vehicle control (0.2% (*v*/*v*) ethanol in HBSS). Chl catabolite stocks were dissolved in ethanol. For PxB when both ethanol and DMSO stocks were analyzed, the type of vehicle did not affect the ROS scavenging potential. Cells were incubated at 37 °C for 1 h. Thereafter, cells were washed twice with PBS. A volume of 90 µL HBSS was filled in each well before the freshly dissolved radical generator was added to achieve a final concentration of 600 µM AAPH. After 30 min incubation at 37 °C the DCF fluorescence was measured at an excitation wavelength of 480 nm and emission wavelength of 535 nm using a Tecan infinite F200 PRO plate reader (Tecan Group Ltd., Männedorf, Switzerland). 

### 2.10. PBMC Treatments and Metabolite Measurements

Human PBMC were seeded in 48-well plates (1 *×* 10^6^ cells/980 µL/well) in a standard culture medium or in a medium containing increasing concentrations of PxB (0.18–45 µM) or vehicle controls (0.01, 0.05 and 0.1% (*v/v*) DMSO), respectively. Six wells were seeded for each treatment condition. After 30 min, either 20 µL of medium or 20 µL of red kidney bean *Phaseolus vulgaris*-derived phytohemagglutinin (PHA) were added to three wells of each condition to achieve a final concentration of 1 mg/mL. After 48 h of incubation, the accumulated concentrations of Kyn, as well as of Trp, were determined in the cell supernatant by HPLC, using 3-nitro-L-tyrosine as an internal standard according to the method described by Widner et al. with minor modifications [35,44]. To estimate the activity of IDO-1 [45], Kyn/Trp was calculated and expressed as μmol Kyn/mmol Trp. The metabolic activity of cells was assessed in one well for each condition by transferring 100 µL of the cell suspension in triplicates to a 96-well plate and addition of 10% (*v*/*v*) of the resazurin-based CellTiter-Blue^®^ redox dye. Viable cells reduce resazurin into resorufin. The fluorescence of resorufin was determined after 4 h of incubation at 560 nm excitation/590 nm emission using the Tecan infinite F200 PRO plate reader. 

### 2.11. Statistical Analysis

Statistical analysis was performed using non-parametric methods, as not all samples showed normal distribution (Wilcoxon Signed Ranks and Friedman) using IBM SPSS Statistics version 21. Differences were considered to be of significance if *p* values were ≤0.05.

## 3. Results

For investigating biologically relevant properties of PBs, we isolated a PleB and its corresponding oxidation product from senescent leaves of *Cercidiphyllum japonicum* and a DPleB from senescent leaves of grapevine (*Vitis vinifera*) according to the published protocols [20,29]. The antioxidative properties of the test substances were investigated in a cell-free approach as well as in living cells, and the most active compound was further analyzed for immunomodulatory properties by targeting Trp metabolism along the Kyn axis. 

### 3.1. Antioxidative Properties of Phyllobilins 

**Figure 1:**
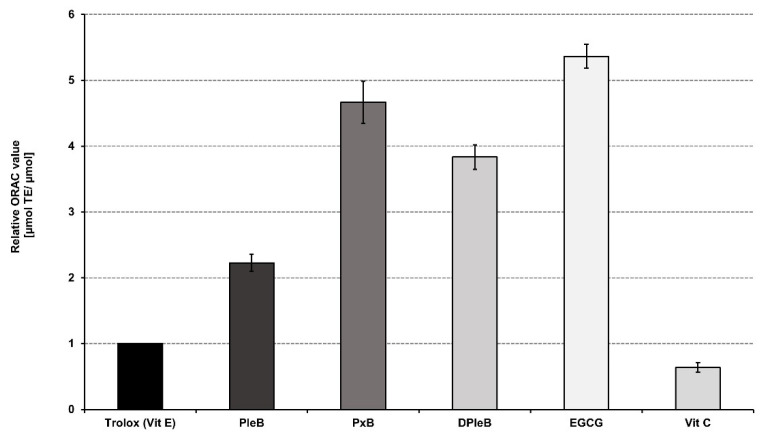
Oxygen radical absorbance capacity (ORAC) of phyllobilins. The capacity to inhibit peroxyl-radical-induced fluorescein oxidation was determined in an *in chemico* assay system, comparing the phyllobilin’s activity with the scavenging potential of Trolox (a water-soluble vitamin E). The relative ORAC value is expressed as µmol Trolox equivalent/µmol test substance. Measurements were preformed 4-times in duplicates (2-times in duplicate for vitamin C (Vit C)), values shown are mean ± standard error of the mean (SEM). EGCG: epigallocatechin-gallate.

The antioxidative capacity of the PBs was evaluated using the oxygen radical absorbance capacity (ORAC) assay, in which a compound’s potential to inhibit peroxyl-radical-induced fluorescein oxidation is determined by taking into account the degree and time of inhibition, thus providing a measure of hydrophilic chain-breaking antioxidant capacity against peroxyl radicals [41]. In this cell free *in chemico* approach, all three PBs demonstrated potent peroxyl-radical scavenging capacity with PxB being most active, resulting in an ORAC value (± standard error of the mean (SEM)) of 4.67 ± 0.32 Trolox equivalents (TE, Figure 1, Supplementary Information, Table S1). For comparison, vitamin C (L-ascorbic acid) is less active than the water-soluble vitamin E analogue Trolox -the resulting ORAC value for vitamin C was 0.64 ± 0.07 TE. 

The antioxidant potential of the PBs in living cells was analyzed by assessing relative changes of intracellular reactive oxidative species (ROS) levels in Caco-2 cells that were monitored by using 2′,7′-dichlorofluorescein diacetate (DCFH-DA) as a substrate. ROS formation was stimulated by addition of the peroxyl radical generator AAPH. In addition to the antioxidant activity of the test compound itself, the cellular antioxidant activity (CAA) assay also accounts for aspects of uptake, metabolism, and location of antioxidant compounds within cells [38]. Briefly, DCFH-DA diffuses through cell membranes and is hydrolyzed by intracellular esterases to non-fluorescent DCFH, which is then trapped within the cell. In the presence of ROS, DCFH is rapidly oxidized to highly fluorescent DCF, whose intensity is proportional to the amount of intracellular ROS. Antioxidants can prevent oxidation of DCFH.

**Figure 2:**
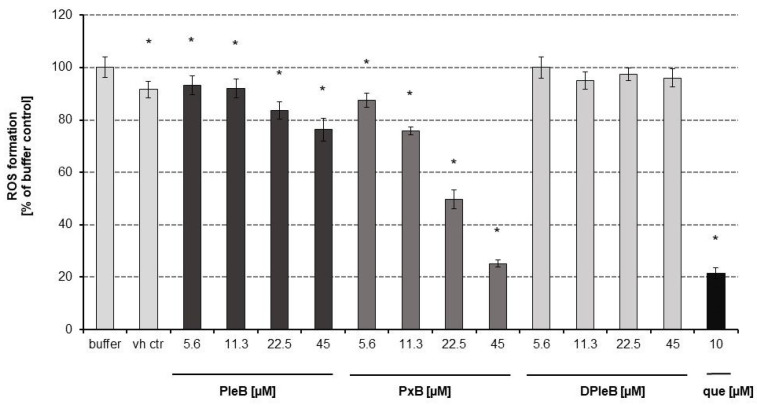
ROS formation in Caco-2 cells. Inhibition of peroxyl radical-induced formation of ROS in cells treated with either quercetin (que, 10 µM), increasing concentrations of PBs PleB, PxB, DPleB (5.6 to 45 µM) or vehicle control (vh ctrl, 0.2% (*v*/*v*) EtOH in HBSS buffer). The mean percentages of DCF fluorescence, as a measure of ROS formation, are shown in relation to the buffer control (set to 100%). Results shown are means ± SEM of at least three independent experiments. * *p* values ≤ 0.05 were considered as significant.

ROS levels were slightly decreased in AAPH-stimulated Caco-2 cells upon treatment with the PleB in a concentration-dependent manner and in comparison to the vehicle control. The incubation with PxB resulted in a dose-dependent reduction of ROS action of up to almost 80% at the highest concentration (45 µM), similar to the effect of the known potent antioxidant quercetin (10 µM). The addition of the DPleB showed no significant inhibition of ROS formation at indicated concentrations (Figure 2). 

### 3.2. Impact of PxB on Tryptophan Breakdown in Human PBMC

Having established that the PxB shows the most prominent effects in attenuating cellular ROS formation and having confirmed that the PxB is stable in cellular media by HPLC analysis (Supplementary Information, Figure S1), its impact on inflammation-induced Trp metabolism was investigated. 

**Figure 3:**
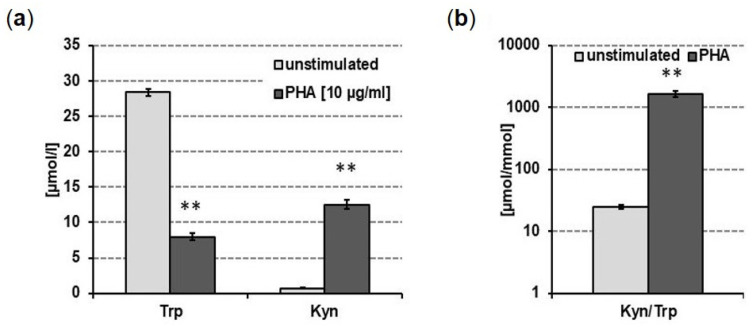
Comparison of (**a**) Trp, Kyn and (**b**) Kyn/Trp concentrations in the supernatants of unstimulated (light grey) and PHA-stimulated (dark grey) PBMC after 48 h of incubation. ** *p* < 0.005 indicates significance compared to the unstimulated cells. Results shown are mean values ± SEM of 6 independent experiments (6 donors) performed in duplicates.

The effect of PxB [0.18–45 µM] on PHA-induced Trp breakdown to Kyn was determined in the supernatants of unstimulated and PHA-stimulated PBMC after 48 h of incubation. The standard culture medium contained a mean Trp concentration of approximately 37 µM. After PHA stimulation, Trp concentration decreased and Kyn levels increased, as reflected by an elevated Kyn/Trp ratio (Figure 3). The mitogen PHA is known to stimulate the release of IFN-γ, TNF-α, and soluble cytokine receptors, to activate related immunobiochemical pathways, such as Trp catabolism and neopterin formation, as well as to induce proliferative responses in PBMC of healthy individuals [46,47,48]. Hence, this cell model reflects various pro- and anti-inflammatory cascades that are of relevance in inflammation; moreover, this activation was previously shown to be sensitive to the activity of antioxidants [35]. 

**Figure 4:**
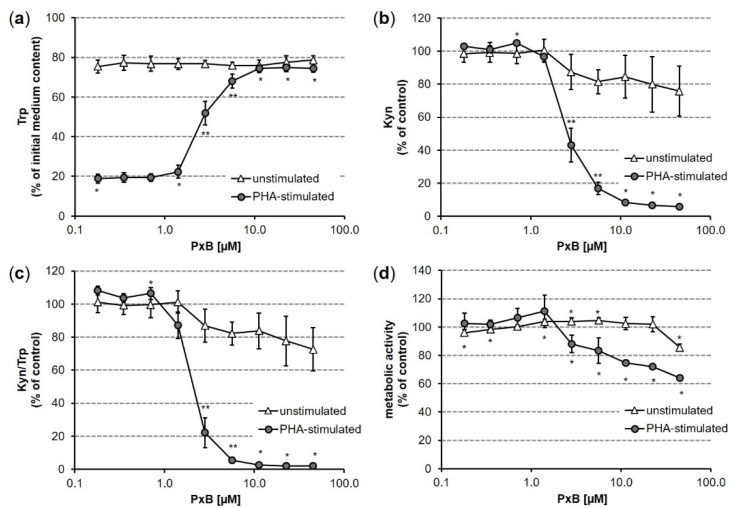
Effect of PxB on tryptophan metabolism. Treatment of phytohemagglutinin (PHA) [10 µg/mL]-stimulated (black circle) and unstimulated (white triangle) PBMC with different concentrations of PxB [0.18–45 µM]. (**a**) Trp concentrations are expressed as [%] of medium control. (**b**) Kyn concentrations, (**c**) the Kyn to Trp ratio (Kyn/Trp), (**d**) and metabolic activity indicated by resazurin conversion, were determined in cell culture supernatants after 48 h of incubation. Values are normalized to vehicle control treatments (set to 100%). Results shown are the mean values ± SEM (* *p* < 0.05, ** *p* < 0.005, compared to baseline).

By addition of PxB [0.18–45 µM] to PHA-stimulated PBMC, Trp concentrations increased in the supernatants in a dose-dependent manner, starting at a 1.4 µM treatment concentration (Figure 4a). This effect was accompanied by a decrease in cell viability (Figure 4d). Treatment with 11.3 µM PxB attenuated Trp consumption, and levels in the supernatants were comparable to unstimulated controls (approximately 75% of initial medium content after 48 h of treatment). On the contrary, Kyn concentrations decreased in these supernatants which resulted in a decreased Kyn/Trp (Figure 4b,c).

In the supernatants of unstimulated cells no effect of PxB treatment on Trp concentrations could be observed, and Kyn concentrations were reduced slightly but mostly not significantly to 80% compared to unstimulated controls (Figure 4a,b). This resulted in a reduced Kyn/Trp reaching a significant maximum of 27% reduction compared to control at a treatment concentration of 45 µM. At this concentration, the first suppressive effect on cell viability was observed (Figure 4c,d).

## 4. Discussion

Detoxification and nutrient recycling are regarded as the primary reasons for the degradation of the green plant pigment [49]. The release of Chl from its binding proteins and the cell membrane is necessary for the remobilization of proteins and lipids in the chloroplast. Free Chl, however, is phototoxic for the cell and needs to be degraded. Mutants that lack early phase degradation enzymes such as PaO and RCCR lead to early cell death phenotypes, which arise from the accumulation of toxic intermediates of the early Chl degradation pathway [50]. Phyllobilins, the end products of this detoxification process, were first thought to be mere waste products. More recent studies, however, suggest a number of significant roles for Chl catabolism in plants [49], e.g., in plant defense; invasion by pathogens has been shown to trigger Chl breakdown, and it is speculated that Chl degradation is linked to the defense of plants against pathogens. A recent publication investigating PBs in sweet basil (*Ocimum basilicum*) [51] as well as a study by Mittelberger et al. on phytoplasma infected apricot and apple trees, showed that pathogen-induced chlorosis also proceeds via the PaO/phyllobilin pathway [52]. Furthermore, phyllobilins, in particular PxBs, are structurally related to bilirubin and phycocyanobilin, both of which have been the focus of recent research, since more and more bioactivities are being discovered for those two molecules. For example, bilirubin (BR), the final product of heme degradation in humans, is known to play a role in the prevention of heart conditions [53]. Phycocyanobilin, found in the blue-green algae spirulina, is a potent anti-inflammatory agent [54]. Both molecules are known to inhibit NADPH oxidase activity [55,56]; the overactivity of this enzyme complex plays a role in many physiological conditions, e.g., vascular disorders, neurodegenerative disorders, and hepatic fibrosis [57,58,59]. Therefore, downregulation of NADPH oxidase has high potential in therapeutic and preventive medicine.

First insights into the bioactivities of PBs revealed the less polar NCC (PleB) from apple and pear to exhibit antioxidative activity in an autoxidation study determining the formation rate of hydroperoxides of linoleic acid, to a similar extent as for BR [60]. On the basis of these promising results for PleBs, previous research focused on the antioxidative properties of PxBs, the products of an oxidation process from PleB precursors, were considered to possess an even higher potential as antioxidants [61]. Indeed, PxBs isolated from the medicinal plant *Echinacea purpurea* were shown to be potent antioxidants in an in vitro assay, as well as to scavenge the hydrogen peroxide-induced ROS production in an intracellular approach in HeLa cells. Measuring intracellular GSH levels also revealed the PxBs to be able to prevent oxidative stress. Furthermore, the uptake of three exemplary PxBs in HEK cells was assessed, demonstrating PxBs to enter the cell membrane and being stable in a cellular environment for at least 4 h [26]. Interestingly, recent research elucidated the cytoskeletal protein actin as a common cellular target of PBs and bilins [62], which underlines the importance of in depth investigations of physiological roles and effects of this class of ubiquitous natural products. 

To obtain a deeper understanding of the biologically relevant properties of PBs, and to directly compare different types of PB structures regarding their antioxidative potential, we selected three PBs with a very simple and identical structure in terms of peripheral modifications of the tetrapyrrole core: one type-I PleB and one dioxobilin-type (or type-II) DPleB from two known branches of the PaO/phyllobilin pathway, as well as the commonly identified oxidation product of the PleB, the PxB. Although PleBs as well as PxBs have been previously reported as antioxidants, we aimed to directly compare the antioxidative potential of the PleB and PxB isolated from the same plant and only differing in a double bond between C15 and C16, as well as the DPleB, carrying a lactam group at ring A instead of a formyl moiety. 

Investigations on biomedical-relevant activities of the type-II class of PBs still lag behind. There are several hints for the possible antioxidant activity of such compounds as type-II PBs resemble the antioxidative catabolites from heme degradation even more than the type-I PBs due to their 1,19-dioxobilin type structure lacking the aldehyde functionality [63]; their potential to influence cellular ROS formation, however, is not well understood. 

Here, we showed that the PBs PleB, DPleB and PxB are active in the ORAC assay, which refers to the direct effects of chain-breaking antioxidants based on the hydrogen atom transfer mechanism. In this cell-free system, PxB was most active. Moreover, the PleB and PxB dose-dependently dampened ROS formation in the Caco-2 cell model, whereas the DPleB was not able to affect intracellular ROS generation. Likewise, in the cell-free system, the PxB turned out to possess the highest antioxidative activity. As mentioned before, the CAA assay accounts not only for antioxidative properties but points also towards uptake properties. Accordingly, uptake of the DPleB and PleB might be limited whereas the PxB seems to be taken up by Caco-2 cells. These data extend the previously published findings regarding cellular uptake of the PleB and PxB from *Cercidiphyllum japonicum*, which revealed both PBs to be taken up by HEK cells but in contrast to the PxB, the PleB assimilated in only minor amounts [29]. 

Going one step further, PBMC were applied to assess IDO-1 activity [35], revealing potential anti-inflammatory properties for the PxB. The IDO-1 stimulated catabolism of Trp belongs to the most essential regulatory immunometabolic pathways of the cellular immune response [32]. The activity of this pathway, often indicated by the Kyn/Trp ratio determined in serum or plasma of patients, is of high relevance as biomarker in several human disorders associated with an activated immune system such as infections and malignancies [37]. PBMC consist mainly of T-cells as well as of B-cells, NK-cells and monocytes/macrophages and thus represent a co-culture system that allows the investigation of T-cell/macrophage interplay in vitro. As mentioned above, critical cascades that are involved in inflammatory responses can be mimicked in this cell model. Moreover, it is a well-established tool to screen for immunomodulatory activities of phytochemicals [34,35].

The dose-dependent effect of PxB in suppressing PHA-induced Kyn formation significantly at low micromolar concentrations, while not affecting unstimulated PBMC, indicates possible anti-inflammatory properties of the substance. So far, it remains unanswered if the attenuation of ROS deriving from the PHA-induced response in the PBMC model is the only target of this compound. Further studies are clearly warranted to clarify whether other gene regulatory or enzyme activity modulatory effects contribute to this effect.

Since ancient times, plant-derived remedies are an integral part of traditional medicinal systems, where they have been used as therapeutic interventions for several inflammatory conditions such as pain, fever, and infections. Still, plant-constituents and botanical extracts are frequently used for the treatment of different clinical conditions, ranging from common cold to severe chronic diseases [64]. Our results raise the question to which extent Chl catabolites may contribute to the pharmacological profiles of botanical remedies, which has practical consequences in terms of quality control of raw materials or importance of harvesting time. 

The number of studies on cell biological effects of PBs is increasing only recently [26,29,62,63], thus bringing a new, previously ignored group of substances in the focus of pharmacological research.

The story of the breakdown products of the green plant pigment Chl started about 30 years ago with the identification of a non-colored PleB, named *Hv*-NCC-1 [5]. PleBs were first thought to be ‘final’ Chl breakdown products, until in 2008 the first PxB could be identified [22]. The mechanism behind the formation of PxBs is not yet fully clarified, however, an ‘oxidative activity’ was detected and proposed to account for the oxidation of PleBs, thus yielding colored, more lipophilic structures [39]. PxBs are increasingly gaining attention not only due to their interesting physicochemical properties [40,65]. PxBs have recently been found to be wider distributed in the plant kingdom than previously assumed and were shown to exceed their structural precursors regarding bioactivities such as anti-cancer effects [29]. We here provide further evidence that, as concerns physiologically relevant activities, the PxBs clearly surpass the PleBs. Thus, bioactive potential, as well as photoactive properties and decreased polarity of PxBs, all strengthen the consideration of Chl breakdown as being more than just producing ‘waste’ products. 

## 5. Conclusions

We herein analyze the in vitro antioxidative activity of a PleB, a DPleB, and a PxB; the latter was shown to be the most potent antioxidant in a cellular approach. Furthermore, we characterized the bioactive properties of the PxB in more detail and found anti-inflammatory activities by suppressing a key metabolic pathway of the Th1-type immune response in human blood-peripheral mononuclear cells. PBs are ubiquitous in the plant kingdom, not only occurring in senescent leaves with visible signs of Chl breakdown but are becoming more and more appreciated as phytochemicals in human nutrition or as ingredients of herbal remedies. With this study, we add a missing piece to the exciting puzzle of the role of PBs in plants as well as in humans, and point towards a large potential for PBs, especially PxBs, as beneficial natural products. 

## Data Availability

Not applicable.

## Supplementary Materials

The following supporting information can be downloaded at: https://www.mdpi.com/article/10.3390/antiox11102056/s1, Figure S1: HPLC traces of PxB treated Caco-2 cells; Table S1: Test concentrations and netAUC in the ORAC assay.
