# Supplementary material for: A Chlorophyll-Derived Phylloxanthobilin Is a Potent Antioxidant That Modulates Immunometabolism in Human PBMC
Source: Antioxidants (Basel). 2022 Oct 19;11(10):2056. doi: 10.3390/antiox11102056 (PMC9599000; doi:10.3390/antiox11102056)
Supplement: Supplementary file 1 [file antioxidants-11-02056-s001.zip › antioxidants-1939388-supplementary.pdf]

A Chlorophyll Derived Phylloxanthobilin is a Potent Antioxidant that modulates Immunometabolism in Human PBMC

Cornelia A. Karg [1], Lucia Parrakova [2], Dietmar Fuchs [3], Harald Schennach[4], Bernhard Kräutler[5], Simone Moser [1,*] and Johanna M. Gostner [2,*]

[1] Department of Pharmaceutical Biology, Ludwigs-Maximilian University of Munich, Butenandtstr. 5-13, 81977 Munich, Germany
[2] Institute of Medical Biochemistry, Medical University of Innsbruck, Innrain 80, 6020 Innsbruck, Austria
[3] Institute of Biological Chemistry, Medical University of Innsbruck, Innrain 80, 6020 Innsbruck, Austria
[4] Central Institute of Blood Transfusion and Immunology, University Hospital, Anichstr. 35, 6020 Innsbruck, Austria
[5] Institute of Organic Chemistry and Center for Molecular Biosciences, University of Innsbruck, Innrain 80/82, 6020 Innsbruck, Austria
* Correspondence: simone.moser@cup.uni-muenchen.de (S.M.); johanna.gostner@i-med.ac.at (J.M.G.); Tel.: +49-89-2180-77175 (S.M.); +43-512-9003-70120 (J.M.G.)

Supplementary Information

Table of content

Supplementary Figures

1. Figure S1 HPLC traces of PxB treated Caco-2 cells …….…………………………………….2
2. Table S1 Test concentrations and netAUC in the ORAC assay…….…………………...…2

Supplementary Figures

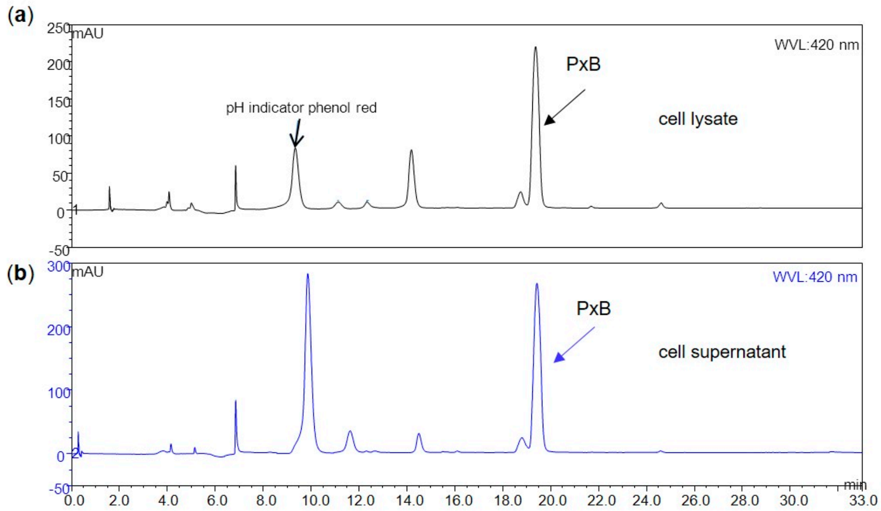

Figure S1. HPLC traces (detected at 420 nm) of PxB treated Caco-2 cells; (**a**) cell supernatant and (**b**) cell lysate were analysed (Gradient 17-45 % ACN in 20 min, 10 mM NH4OAc buffer, 500 µl/min).

sample	µmol TE/µmol compound	+/- SEM	concentration range measured [µM]	slope	intercept	R2
**Trolox (Vit E)**	**1.00**		0.78 - 6.25	3.5012	1.0977	0.9960
**PleB**	**2.23**	**0.13**	0.30 - 2.39	7.0909	1.7436	0.9784
**PxB**	**4.67**	**0.32**	0.19 - 1.54	11.7790	3.6418	0.9607
**DPIeB**	**3.83**	**0.19**	0.24 - 1.95	9.1142	4.1643	0.9444
**EGCG**	**5.36**	**0.18**	0.17 - 0.68	18.6660	2.0802	0.9870
**Vit C**	**0.64**	**0.07**	0.44 - 3.55	0.4938	0.3189	0.9861

Table S1. Test compound concentrations and netAUC in the ORAC assay: A dose-dependent increase of the fluorecein rescue from oxidative decay in the the oxygen racical absorbance capacity assay (ORAC) is indicated by a linear increase of the netAUC (AUC = area under curve; netAUC = AUCsample – AUCblank), as shown by the slope and intercept of the straight line, and the regression coefficient (R^2). The final concentration range of the test compounds in the assay mixture is listed. Results shown are derived from four independent measurements.
